# *Haemophilus parainfluenzae* as a Causative Organism in Periprosthetic Joint Infection: A Case Report

**DOI:** 10.1155/crdi/6158663

**Published:** 2026-08-28

**Authors:** Vincent K. Melemai, Ryan J. Blake, Adam E. Klein, Allison M. Lastinger

**Affiliations:** ^1^ Department of Orthopaedics, West Virginia University, P. O. Box 9196 64 Medical Center Drive, Morgantown 26506-9196, West Virginia, USA, wvu.edu; ^2^ Department of Medicine, West Virginia University, P. O. Box 9163 64 Medical Center Drive, Morgantown 26506-9163, West Virginia, USA, wvu.edu

**Keywords:** case report, *Haemophilus*, periprosthetic joint infection

## Abstract

**Background:**

*Haemophilus parainfluenzae* (*H. parainfluenzae*) is rare in periprosthetic joint infections (PJIs). While many cases are associated with a prior history suggestive of an oral source, many occur without an identifiable source or exposure. We review two patients who had concerns for PJI, later identified as *H. parainfluenzae* through intraoperative cultures.

**Case Report:**

Two patients presented with complaints of knee pain. The first, a 54‐year‐old male, was found on admission to have a knee PJI. The patient underwent debridement, antibiotics, and implant retention (DAIR) and was discharged on intravenous (IV) ceftriaxone after *H. parainfluenzae* was isolated on culture, with no resistance reported on susceptibilities. However, approximately 4 weeks later, the patient underwent a second DAIR due to persistent swelling and redness; cultures were negative at the time of the second surgery. He was treated with IV vancomycin and cefepime and placed on cefadroxil suppression therapy without additional surgery. The second patient, an 80‐year‐old male, had a history of prior knee PJI, methicillin‐resistant *staphylococcus aureus* (MRSA) in 2009 and *Streptococcus agalactiae* in 2015. He presented with a draining sinus and pain over his knee. Resection arthroplasty and above knee amputation were offered, but he would only consent to DAIR; intraoperative cultures showed *H. parainfluenzae*. He was treated with IV ceftriaxone postoperatively and then oral suppression.

**Conclusions:**

Due to increasing resistance in this species worldwide, consideration should be taken when choosing antimicrobial therapy for *H. parainfluenzae*. From the limited data available, patients with PJI secondary to *H. parainfluenzae* have a better prognosis compared to PJI with other Gram‐negative organisms.

## 1. Introduction

The genus *Haemophilus,* a member the HACEK (*Haemophilus, Aggregatibacter, Cardiobacterium, Eikenella, and Kingella*) group of organisms, is characterized by a collection of Gram‐negative coccobacilli that share similar properties in terms of in vitro growth requirements and related structural features. Metabolism requires the presence of a single substance or a combination of substances related to erythrocytes, most notably hemin on the cell membrane and nicotinamide adenine dinucleotide (NAD+) confined intracellularly [1]. *Haemophilus* bacteria also contain a smaller lipooligosaccharide on their cell surface that functions similarly to lipopolysaccharide common to many other Gram‐negative organisms [1]. The *H. influenzae* subtype, specifically, grows only in the presence of both hemin and NAD+ and represents a majority of *Haemophilus* infections with further subcategorization into typeable and nontypeable lineages dependent on capsular polysaccharide antigens [1]. Other species include *H. parainfluenzae*, *H. aegyptius,* and *H. ducreyi* that can cause cardiopulmonary infection, conjunctivitis purpuric fever, and chancroid, respectively [1].

*H. parainfluenzae* has major implications in the development of pneumonia and endocarditis [2], but many reports have highlighted the pathogen’s ability to cause infection of the genitourinary tract [3]. This bacterium typically resides in the oropharynx, which corresponds to its role in the development of upper and lower respiratory tract infections [4]. However, residence in the genitourinary system potentially provides a spatial advantage for causing infections unrelated to the respiratory tract.

Other bacterial organisms such as coagulase‐positive (*Staphylococcus aureus*) and coagulase‐negative *Staphylococcal* species, *Streptococcal* species, Gram‐negative bacilli, and enterococcus contribute most frequently to surgical infections [5]. Gram‐negative bacteria cause up to 20% of periprosthetic joint infections (PJIs) and pose greater treatment challenges due to biofilm formation and antimicrobial resistance, resulting in higher failure rates compared to Gram‐positive organisms [6]. Common pathogens include *Pseudomonas aeruginosa*, Enterobacterales, and occasionally organisms such as *Haemophilus* species, with their identification being clinically important as both antimicrobial and surgical management may differ substantially from Gram‐positive PJI [7].

*Staphylococcus epidermidis*, coagulase‐negative *Staphylococci*, characteristically affects non‐native, prosthetic joints and related hardware due to its unique ability to bind foreign material with subsequent production of a biofilm [8]. The gelatinous structure contains polysaccharides resistant to host defenses and many antibiotic therapies. Due to its prevalence among reported PJI cases, empiric therapy ensures targeted treatment of the Gram‐positive cocci [9]. However, only a small number of reports in the literature have described *H. parainfluenzae* as the causative agent in joint infection, and an even smaller number describe PJI specifically [10]. Due to its rarity, epidemiological studies have not been published concerning *H. parainfluenzae* in PJI. Herein, this case report reviews two patients who presented to a tertiary referral center for treatment of PJI secondary to *H. parainfluenzae*.

## 2. Case Reports

### 2.1. Case #1

A 54‐year‐old male with a history of multiple surgeries on the left knee, most recently being a revision of a left total knee arthroplasty 2 years prior, presented with a 4‐day history of left knee pain, swelling, and warmth concerning for left knee PJI. On admission, aspiration of the joint revealed 107,500 cells/μL white blood cells (WBCs), 87% polymorphonuclear neutrophils (PMNs), 16,000 cells/μL red blood cells (RBCs), and 3+ leukocyte esterase positivity. The patient’s erythrocyte sedimentation rate (ESR) was 25 mm/h, and C‐reactive protein (CRP) was 143.7 mg/L, increasing to a maximum of 283.4 mg/L prior to operative intervention. However, the patient remained afebrile, and blood cultures remained negative throughout the hospitalization. The patient underwent a debridement, antibiotics, and implant retention (DAIR) procedure and was placed on empiric vancomycin and cefepime while intraoperative cultures were pending. All five intraoperative cultures demonstrated positivity for *H. parainfluenzae,* showing susceptibility to ceftriaxone and negative beta‐lactamase activity (Table 1). At that time, the patient was discharged with a 6‐week course of intravenous (IV) ceftriaxone with subsequent oral cefadroxil suppression planned. However, 10 days later, the patient developed increased swelling, erythema, and warmth over the left knee. He was seen in clinic, where repeat aspiration showed 10,500 cells/μL WBC, 93% PMN, 250,000 cells/μL RBC, and 2+ leukocyte esterase positivity with no growth for 14 days. Serum laboratory studies revealed an ESR of 60 mm/h and a CRP of 74.5 mg/L at that time. As the patient was still actively being treated with IV ceftriaxone, the decision was made to continue the course of antibiotics, and the patient was instructed to return if the joint acutely worsened.

**TABLE 1:** Antimicrobial susceptibilities, case 1.

Antibiotic	MIC (units = μg/mL) and interpretation
Beta‐lactamase	Negative
Meropenem	≤ 0.06, susceptible
Azithromycin	4, susceptible
Ciprofloxacin	≤ 0.12, susceptible
Levofloxacin	≤ 0.06, susceptible
Trimethoprim/sulfamethoxazole	≤ 0.12/2.4, susceptible
Cefuroxime	0.5, susceptible
Ampicillin	0.25, susceptible
Ceftriaxone	≤ 0.06, susceptible

Abbreviations: MIC, minimum inhibitory concentration; NR, not reported.

Sixteen days later, the patient presented to the emergency department for worsening knee pain, edema, and erythema but remained afebrile. Repeat aspiration was performed showing 2625 cells/μL WBC, 91% PMN, 272,500 cells/μL RBC, and 2+ leukocyte esterase positivity. Serum ESR was 41 mm/h, and CRP was 92.7 mg/L at that time. Computed tomography (CT) showed no periprosthetic lucency but did demonstrate a large peripherally hyperattenuating collection, which was suspicious for an abscess and a moderate‐to‐large left knee effusion. Blood cultures were drawn and later found to be negative. Operative options were discussed with the patient, and given the stability of the implant and the patient’s comorbidities, a shared decision was made to proceed with a second DAIR with an extended course of postoperative antibiotic. This treatment plan would ideally preserve a well‐functioning, well‐fixed arthroplasty and avoid the morbidity of an explant or amputation, although the high chance of failure was addressed with the patient. Two days later he was taken for a repeat DAIR of the left knee with extensor mechanism repair. Joint cultures remained negative throughout admission, and the patient was treated with vancomycin and cefepime with a plan to treat with dual antibiotic therapy for 6 weeks. The patient completed the 6 weeks of planned outpatient IV antibiotics and was transitioned to cefadroxil with seemingly successful suppression.

An 80‐year‐old male with a complex surgical history regarding his right knee with multiple previous PJI caused by methicillin‐resistant *Staphylococcus aureus* (MRSA) and *Streptococcus agalactiae* presented to our center with concern for PJI in his right knee. Nine years prior, the patient underwent an explant of the right knee with placement of a static antibiotic spacer and completed 6 weeks of vancomycin and ertapenem followed by reimplantation of the right knee. He remained asymptomatic with no complaints concerning the knee until he experienced multiple lesions over the knee with development of a draining sinus, erythema, and edema, which first started 2 months prior to presentation. Prior to arrival, he was treated at an outside hospital with a single dose of clindamycin after laboratory studies revealed a WBC count within normal limits with plain radiograph showing lucency between the tibial component of the arthroplasty and medial tibial bone.

Upon arrival to our center, he received a CT scan of the right leg which showed skin thickening with questionable fluid collection at the medial aspect of the right knee and lucency surrounding the tibial hardware. The orthopedic surgery team performed an aspiration of the right knee; however, the fluid clotted, and a cell count could not be performed. There was trace leukocyte esterase positivity, and the sample was sent for culture. Blood cultures were obtained and ultimately returned negative. Prior to operative intervention, ESR and CRP were found to be elevated at 67 mm/h and 54.0 mg/L, respectively.

Given the patient’s draining sinus and fully cemented long femoral and tibial stems, an above‐the‐knee amputation was recommended; however, the patient declined an amputation and consented only to a washout. Therefore, the patient underwent a DAIR procedure, and the patient was started on daptomycin and cefepime postoperatively. All five intraoperative cultures ultimately grew *H. parainfluenzae*, and susceptibilities were obtained (Table 2). The patient was transitioned to IV ceftriaxone, and a 6‐week postoperative course was planned prior to discharge.

**TABLE 2:** Antimicrobial susceptibilities, case 2.

Antibiotic	MIC (units = μg/mL) and interpretation
Beta‐lactamase	Negative
Meropenem	≤ 0.06, susceptible
Azithromycin	NR
Ciprofloxacin	NR
Levofloxacin	NR
Trimethoprim/sulfamethoxazole	NR
Cefuroxime	NR
Ampicillin	0.25, susceptible
Ceftriaxone	≤ 0.06, susceptible

Abbreviations: MIC, minimum inhibitory concentration; NR, not reported.

## 3. Discussion

Although many cases in the literature of bone and joint infection caused by *H. parainfluenzae* were successfully treated with a penicillin or cephalosporin, resistant and multidrug‐resistant strains of *H. parainfluenzae* have been isolated with increasing prevalence, which highlights the potential for increasing pathogenicity [11]. *H. parainfluenzae* has been found to be the causative agent for both native and PJIs, though it remains a rare cause of infections. It is generally associated with the oral cavity after dental procedures in addition to cardiopulmonary infections [1, 10, 12]. However, in many of these cases, no risk factor for infection was identified, creating a challenging scenario dependent on high clinical suspicion [12]. Neither patient could recall a dental procedure or respiratory infection preceding the development of their symptoms. While both patients examined within this case report were male, with one of advanced age, there is currently insufficient evidence to establish sex and age as independent risk factors for *Haemophilus* PJI susceptibility. Larger studies are needed to determine whether this demographic pattern reflects a true association or publication bias.

Presentation varies among patients with *H. parainfluenzae* native joint infections, though pain was reported to be the most common presenting symptom even in the absence of inflammatory signs [12]. Among these patients, joint aspirate WBC count was not a sensitive predictor of infection; however, ESR and CRP levels were generally elevated in these patients [12]. Generally, these patients have favorable outcomes, though the duration of their antimicrobial therapy may be prolonged prior to resolution of infection [12].

Although not classically associated with biofilm forming PJI, a strain of *H. parainfluenzae* has been shown to form in vitro biofilms [13], which have been demonstrated to contribute to persistent joint fluid culture‐negative PJI [14]. Similarities can be drawn between this finding and *Haemophilus’* classification as a cause of culture‐negative endocarditis. The difficulty in culturing the Gram‐negative organism appears to span multiple infection locales. Although resistance patterns vary greatly between regions, third and fourth generation cephalosporins are generally effective against most strains of *H. parainfluenzae*, despite the growing prevalence of beta‐lactamase–producing strains [15].

In patients with biofilm‐related PJI, duration of antimicrobial therapy differs based on the operative approach, with therapy duration ranging from three to 6 months for DAIR and four to 6 weeks for other procedures, although studies differ on optimal duration [16]. While rates of *H. parainfluenzae* associated PJI are reportedly low, underreporting may contribute to the low prevalence due to microbial sensitivity to empiric therapy. Both patients successfully retained their prostheses with surgical debridement and medical management focusing on cephalosporin treatment for infection. Due to the susceptibilities of the *H. parainfluenzae* strains presented, antibiotic treatment following orthopedic intervention was optimized at 6 weeks. However, as previously stated, this timeframe can vary greatly depending on the unique clinical case and bacterial characteristics.

## 4. Conclusion

With a paucity of literature describing the rates of recurrence, outcomes, and complications associated with *H. parainfluenzae* related PJI, consideration of local patterns of resistance of *H. parainfluenzae* should be considered in culture negative PJI where *H. parainfluenzae* cannot be ruled out or where recent dental procedures may have provided a source of infection.

## Funding

This work did not receive any specific grant from funding agencies in the public, commercial, or not‐for‐profit sectors.

## Disclosure

A preprint has previously been published by Melemai et al., on SSRN [17].

## Ethics Statement

Ethics statement was not required for this study.

## Consent

Written informed consent was obtained from the patients presented.

## Conflicts of Interest

The authors declare no conflicts of interest.

## Data Availability

The datasets used and/or analyzed during the current study are available from the corresponding author upon reasonable request.
